# Obesity-associated gene FTO rs9939609 polymorphism in relation to the risk of tuberculosis

**DOI:** 10.1186/s12879-014-0592-2

**Published:** 2014-11-07

**Authors:** Yan Feng, Fengliang Wang, Hongqiu Pan, Sangsang Qiu, Jieqiong Lü, Liang Wu, Jianming Wang, Cheng Lu

**Affiliations:** Division of Parasite Disease Intervention, Zhejiang Provincial Center for Disease Control and Prevention, Zhejiang, China; Department of Epidemiology and Biostatistics, School of Public Health, Nanjing Medical University, Nanjing, China; Department of Breast, Nanjing Maternity and Child Health Hospital of Nanjing Medical University, Nanjing, China; Department of Tuberculosis, Third Hospital of Zhenjiang City, Zhenjiang, China

## Abstract

**Background:**

Obesity is known to affect cell-mediated immune responses. Recent studies have revealed that genetic polymorphisms in the fat mass and obesity associated (*FTO*) gene are related to human obesity. We hypothesize that this gene may also play a role in the risk of immune-related infectious diseases such as tuberculosis.

**Methods:**

This case-control study included 1625 pulmonary tuberculosis cases and 1570 unaffected controls recruited from the Jiangsu province in China. Single nucleotide polymorphisms (SNPs), rs9939609 and rs8050136, in the *FTO* gene were genotyped using TaqMan allelic discrimination assays. Odds ratios (ORs) and 95% confidence intervals (CIs) were calculated using the unconditional logistic regression model.

**Results:**

We observed a significant association between the genetic polymorphism rs9939609 and tuberculosis risk. Compared with the common genotype TT, individuals carrying AA had a significantly increased risk, with an OR of 3.77 (95% CI: 2.26-6.28). After adjusting for potential confounders, the relationship remains significant. An additive model showed that carriers of an allele A had a 26% increased risk of tuberculosis compared with the T allele (OR: 1.26, 95% CI: 1.08-1.48). Compared with the common haplotype rs9939609T-rs8050136C, the haplotype rs9939609A-rs8050136C was related to an increased risk of tuberculosis (OR = 6.09, 95% CI: 3.27-12.34).

**Conclusions:**

The *FTO* polymorphism rs9939609 is associated with a risk of pulmonary tuberculosis in the Chinese population.

## Background

Tuberculosis (TB), a chronic infectious disease caused by *Mycobacteria tuberculosis* and continues to be a major global health threat, particularly in developing countries. In 2012, an estimated 8.6 million new cases occurred, and approximately 1.3 million people died worldwide due to TB (WHO. Global tuberculosis report 2013). Malnutrition, tobacco smoking, immunosuppressive treatment, diabetes, indoor air pollution, crowding, migration, aging and poverty have all been recognized as possible risk factors [1]-[5]. Among these factors, weight-related problems have gained more focus recently because several studies have revealed that obesity is not only associated with malignancies (e.g., breast cancer and colorectal cancer) [6], cardiovascular disorders [7] and type 2 diabetes [8] but is also related to multiple infectious diseases [9].

Until recently, little was known about genetic determinants of obesity, with the exception of a few monogenic, extreme forms [10]. It is estimated that 40% to 90% variation in weight or obesity is attributed to genetic factors, according to twins or adoption studies [11]-[15]. Since then, genome-wide association studies (GWAS) have become available [16], and great advances have been made in exploring the association of genetic polymorphisms with human diseases and traits [17],[18]. The first single nucleotide polymorphism (SNP) robustly related to body mass index (BMI) was mapped to one gene in 2007. This gene, now known as *FTO* (fat mass and obesity associated, Gene ID: 79068), has been repeatedly found to affect obesity in different populations [19]-[22]. The *FTO* gene is located on chromosome 16q12.2, which is well conserved in vertebrate species (e.g., fish and chicken) and plays a role in regulation of food intake [23]. This gene encodes a protein with a novel C-terminal α-helical domain and an N-terminal double-strand β-helix domain. In vitro, the FTO protein can demethylate single-stranded RNA or DNA, but the exact physiological and function is still unknown [24]. The genetic polymorphisms rs9939609 and rs8050136 within the *FTO* gene were found to be significantly associated with obesity in different populations [25]-[28], including the Chinese population [29]-[32].

Recent studies have further shown the effects of the *FTO* gene on the risk of BMI-related outcomes such as diabetes [33], neural tube defects [33] and Alzheimer's disease [34]. Although the effects of obesity on the development of metabolic and cardiovascular problems are well studied, much less is known about its impact on the infectious diseases. Obesity, similar to other states of malnutrition, is known to impair immune function, alter leukocyte counts and affect cell-mediated immune responses [35]. The impairment of the immune function leads to an increased susceptibility of the host to pathogens such as influenza, Mycobacterium tuberculosis, coxsackie virus, Helicobacter pylori and encephalomyocarditis virus [9]. We hypothesize that the *FTO* gene may play an important role in the risk of immune-related human infectious diseases such as tuberculosis. To test this hypothesis, we performed a case-control study to assess the association of two common *FTO* polymorphisms (rs9939609 and rs8050136) with pulmonary tuberculosis in a Chinese population.

## Methods

### Study subjects and data collection

This case-control study included 1625 pulmonary tuberculosis cases and 1570 unaffected controls recruited from Jiangsu province in China. All cases and controls were unrelated Han Chinese population. The cases were recruited from local Centers for Disease Control and Prevention and TB dispensaries. All cases were diagnosed by specialized doctors based on clinical signs, chest x-ray examination, sputum smear tests or sputum culture. Controls were recruited through their participation in local community-based health examination programs. Those with a history of tuberculosis or malignancy were excluded. Controls were frequency matched to cases by sex and age. None of the cases and controls had an HIV-positive history. Trained interviewers administered a questionnaire to collect data on their demographic characteristics, tobacco smoking, alcohol consumption and health care-seeking history. A blood sample was collected from each participant for molecular analysis.

### Genotyping

We extracted genomic DNA from peripheral blood samples by proteinase K digestion and a modified phenol-chloroform protocol. The DNA was frozen at −20°C until genotyping. Genotype determination of rs9939609 and rs8050136 in the *FTO* gene was carried out using TaqMan allelic discrimination assays on the ABI 7900HT Real-Time PCR System (Applied Biosystems, Foster City, CA, USA) and was ascertained using the SDS software (version 2.3). The sequence specific forward and reverse primers and Taqman MGB probes are listed in Table 1. To avoid batch bias, we allocated DNA samples of cases and controls in each plate with no discrepancies between amplification reaction conditions. The overall calling rate was 99.2% for rs9939609 and 98.4% for rs8050136. We randomly selected samples with different genotypes for gene sequencing. The consistent rate was 100%.

**Table 1:** **Primers and probes used to detect SNPs**

SNPs		Sequence (5'-3')	Detecting allele
rs9939609	F	CTAACATCAGTTATGCATTTAGAATGTCTG	
	R	CCCACTCCATTTCTGACTGTTACC	
	Probe-T	FAM-CTGTGAATTT**T**GTGATGC-MGB	Allele T
	Probe-A	HEX-CTGTGAATTT**A**GTGATGCA-MGB	Allele A
rs8050136	F	CAGTGCCAGCTTCATAGCCTAGT	
	R	CCATGAGTCCATCTCTACAGTTTACC	
	Probe-C	HEX-CTGTGGCAAT**C**AATAT-MGB	Allele C
	Probe-A	FAM-CTGTGGCAAT**A**AATA-MGB	Allele A

### Statistical analysis

Data were entered with EpiData 3.1 (Denmark) and analyzed using STATA 10.0 (StataCorp, College Station, TX, USA). Student's t-test (for continuous variables) and the χ^2^ test (for categorical variables) were used to analyze the differences in demographic variables and potential risk factors between cases and controls. Hardy-Weinberg equilibrium (HWE) was tested using a goodness-of-fit χ^2^ test by comparing the observed genotype frequencies with the expected ones among the controls to ensure that the alleles were independently segregated. To evaluate the effects of genetic polymorphisms of the *FTO* gene on the risk of tuberculosis, we calculated odds ratios (ORs) and 95% confidence intervals (CIs) using the unconditional logistic regression model. To avoid potential confounding, we further adjusted by age, sex, tobacco smoking and alcohol consumption. To analyze the effect of SNPs comprehensively, we applied three different genetic models: the additive model, the dominant model and the recessive model. Haplotype analysis on SNP rs9939609 and rs8050136 was performed using the phase 2.1 software.

### Ethical approval

The study was approved by the Institutional Review Board of Nanjing Medical University. Informed consent was obtained for both the interviews and the blood samples.

## Results

The genotype distributions of two SNPs were all in HWE in the controls (P = 0.381 for rs9939609; P = 0.368 for rs8050136). The general characteristics of cases and controls are shown in Table 2. In total, 1583 cases and 1544 controls were successfully genotyped and analyzed. Due to the frequency matching, there was no significant difference in the distribution of both age and sex between the groups. The proportion of ever smokers was 52.64% among cases which was significantly higher than that among controls (34.84%). However, alcohol consumption was inversely related to tuberculosis, where 22.50% of the cases vs. 26.19% of the controls had a history of alcohol consumption (*P* = 0. 017).

**Table 2:** **General characteristics of cases and controls**

Variables	Control (n = 1544)	Case (n = 1583)	***P***
	n (%)	n (%)	
**Sex**			0.304
Male	1118(72.41)	1172(74.04)	
Female	426(27.59)	411(25.96)	
**Age (mean ± SD)**	52.08 ± 17.05	52.13 ± 17.71	0.935
**Tobacco smoking** ^*^			<0.001
Never	1006(65.16)	745(47.36)	
Ever	538(34.84)	828(52.64)	
**Alcohol consumption** ^*^			0.017
Never	1116(73.81)	1209(77.50)	
Ever	396(26.19)	351(22.50)	
**Sputum smear test**			-
Positive	-	1077(68.04)	
Negative	-	506(31.96)	
**Sputum culture**			-
Positive	-	570(36.01)	
Negative or without culture results	-	1013(63.99)	

^*^There are some missing values.

There was a significant association between rs9939609 (T > A) and the risk of tuberculosis. Compared with the common genotype TT, those carrying the AA genotype had a significantly increased risk, with an OR of 3.77 (95% CI: 2.26-6.28). After adjusting for potential confounders (sex, age, smoking and alcohol consumption), the association remained significant [adjusted OR: 3.57 (95% CI: 2.13-6.01)]. As shown in Table 3, allele A carriers had a significantly increased risk among both clinical tuberculosis cases [OR: 1.26 (95% CI: 1.08-1.48), *P* = 0.004] and smear-positive cases [OR: 1.21 (95% CI: 1.02-1.44), *P* = 0.030]. The additive model showed that with each increased number of A alleles, the risk for tuberculosis increased accordingly [crude OR: 1.23 (95% CI: 1.06-1.42), adjusted OR: 1.22 (95% CI: 1.05-1.42)]. Stratified analysis revealed that the effect of AA genotype of rs9939609 on tuberculosis was more evident among men, ever smokers and alcohol drinkers (Tables 4 and 5). However, the heterogeneity wasn't significant across the strata. A significant association between the polymorphisms of rs8050136 and tuberculosis was only observed among never drinkers. Compared with the wild type CC, the OR(95% CI) was 0.22(0.06-0.83) for rs8050136 AA genotype. However, it was not significant after the Bonferroni multiple comparison correction.

**Table 3:** **The association between two SNPs within the FTO gene and the risk of tuberculosis**

SNPs	Control (n = 1544)	Total cases (n = 1583)					Smear-positive cases (n = 1077)				
	n (%)	n (%)	cOR (95% CI)	***P***	aOR (95% CI)	***P***	n (%)	cOR (95% CI)	***P***	aOR (95% CI)	***P***
**rs9939609 (T > A)**											
TT	1250(80.91)	1258(79.52)	1		1		856(79.48)	1		1	
AT	275(17.86)	253(15.94)	0.91(0.75-1.10)	0.316	0.91(0.74-1.10)	0.316	182(16.90)	0.96(0.78-1.18)	0.723	0.96(0.77-1.19)	0.699
AA	19(1.23)	72(4.54)	3.77(2.26-6.28)	<0.001	3.57(2.13-6.01)	<0.001	39(3.62)	2.99(1.73-5.17)	<0.001	2.84(1.61-5.01)	<0.001
T allele	2767(89.84)	2777(87.35)	1				1894(87.93)	1			
A allele	313(10.16)	397(12.51)	1.26(1.08-1.48)	0.004			260(12.07)	1.21(1.02-1.44)	0.030		
Add			1.23(1.06-1.42)	0.006	1.22(1.05-1.42)	0.011		1.20(1.01-1.41)	0.035	1.18(0.99-1.40)	0.059
Dom			1.10(0.92-1.31)	0.296	1.09(0.91-1.30)	0.374		1.10(0.90-1.34)	0.343	1.09(0.89-1.33)	0.424
Rec			3.82 (2.30-6.37)	<0.001	3.64(2.17-6.10)	<0.001		3.02(1.73-5.25)	<0.001	2.86(1.62-5.04)	<0.001
**rs8050136 (C > A)**											
CC	1250(80.91)	1265(79.96)	1		1		863(80.13)	1		1	
AC	282(18.31)	311(19.60)	1.09(0.91-1.30)	0.347	1.08(0.19-1.26)	0.408	210(19.5)	1.08(0.89-1.32)	0.448	1.07(0.87-1.32)	0.513
AA	12(0.78)	7(0.44)	0.58(0.23-1.47)	0.248	0.48(0.19-1.26)	0.136	4(0.37)	0.48(0.16-1.50)	0.209	0.40(0.13-1.28)	0.123
C allele	2774(90.06)	2849(89.76)	1				1936(89.88)	1			
A allele	306(9.94)	325(10.24)	1.03 (0.88-1.22)	0.690			218(10.12)	1.02(0.85-1.23)	0.826		
Add			1.04(0.88-1.23)	0.632	1.02(0.86-1.22)	0.804		1.03(0.85-1.24)	0.789	1.01(0.83-1.22)	0.957
Dom			1.07(0.90-1.28)	0.461	1.05(0.88-1.27)	0.569		1.06(0.87-1.28)	0.590	1.04(0.85-1.28)	0.702
Rec			0.57 (0.22-1.44)	0.234	0.48(0.18-1.24)	0.127		0.48(0.15-1.48)	0.200	0.40(0.12-1.26)	0.117*

Add additive model, Dom dominant model, Rec recessive model, cOR crude odds ratio, CI confidence interval, aOR adjusted odds ratio, adjusted for age, sex smoking and alcohol drinking.

**Table 4:** **The association between two SNPs within the FTO gene and the risk of tuberculosis stratified by sex**

SNPs	Male (n = 2290)			Female (n = 837)		
	n (%)	OR (95% CI)^a^	***P*** ^a^	n (%)	OR (95% CI)^a^	***P*** ^a^
**rs9939609 (T > A)**						
TT	1823(79.61)	1		685 (81.84)	1	
AT	396(17.29)	0.93(0.74-1.16)	0.508	132 (15.77)	0.85(0.58-1.24)	0.397
AA	71(3.10)	4.15(2.23-7.70)	<0.001	20 (2.39)	2.46(0.93-6.47)	0.069
Add		1.27 (1.06-1.51)	0.008		1.09(0.80-1.47)	0.590
Dom		1.13(0.91-1.39)	0.257		0.98(0.68-1.40)	0.893
Rec		4.20(2.27-7.79)	<0.001		2.52(0.96-6.63)	0.061
**rs8050136 (C > A)**						
CC	1827 (79.78)	1		688 (82.20)	1	
AC	448 (19.56)	1.12(0.90-1.39)	0.301	145 (17.32)	0.98(0.68-1.42)	0.924
AA	15 (0.66)	0.53(0.18-1.52)	0.237	4 (0.48)	0.35(0.04-3.38)	0.364
Add		1.06(0.86-1.29)	0.591		0.93(0.66-1.32)	0.692
Dom		1.09(0.88-1.35)	0.413		0.51(0.18-1.49)	0.220
Rec		0.51(0.18-1.49)	0.220		0.35(0.04-3.38)	0.365

^a^adjusted for age, sex smoking and alcohol drinking where appropriate, Add additive model, Dom dominant model, Rec recessive model, OR odds ratio, CI confidence interval.

**Table 5:** **The association between two SNPs within the FTO gene and the risk of tuberculosis stratified by smoking and alcohol drinking**

SNPs	Smoking						Alcohol drinking					
	Never (n = 1751)			Ever (n = 1366)			Never (n = 2325)			Ever (n = 747)		
	n (%)	OR (95% CI)^a^	P^a^	n (%)	OR (95% CI)^a^	P^a^	n (%)	OR (95% CI)^a^	P^a^	n (%)	OR (95% CI)^a^	P^a^
**rs9939609 (T > A)**												
TT	1421( 81.15)	1		1078(78.92)	1		1868 (80.34)	1		594 (79.52)	1	
AT	292(16.68)	0.98 (0.76-1.28)	0.906	235(17.20)	0.81(0.60-1.09)	0.163	394 (16.95)	0.91(0.72-1.13)	0.391	125 (16.73)	0.88(0.59-1.31)	0.536
AA	38( 2.17)	2.87( 1.43-5.77)	0.003	53(3.88)	4.69(2.08-10.59)	<0.001	63 (2.71)	2.42(1.36-4.32)	0.003	28 (3.75)	15.39(3.78-134.54)	<0.001
Add		1.20(0.98-1.48)	0.083		1.23(0.99-1.54)	0.065		1.12 (0.94-1.34)	0.200		1.51 (1.13-2.03)	0.006
Dom		1.11(0.87-1.42)	0.388		1.05(0.80-1.38)	0.720		1.03 (0.83-1.27)	0.799		1.27 (0.88-1.82)	0.196
Rec		2.88(1.44-5.77)	0.003		4.86(2.16-10.96)	<0.001		2.46 (1.39-4.39)	0.002		14.65(3.44-62.34)	<0.001
**rs8050136 (C > A)**												
CC	1428(81.55)	1		1078(78.92)	1		1878 (80.77)	1		591 (77.49)	1	
AC	316(18.05)	1.13(0.88-1.45)	0.336	276(20.20)	1.02(0.77-1.35)	0.888	434 (18.67)	1.05(0.84-1.30)	0.673	150 (20.08)	1.18(0.82-1.69)	0.383
AA	7(0.40)	0.22(0.03-1.81)	0.157	12(0.88)	0.68(0.21-2.17)	0.512	13 (0.56)	0.22(0.06-0.83)	0.025	6 (0.80)	2.12(0.38-11.68)	0.389
Add		1.06(0.83-1.34)	0.644		0.98(0.76-1.27)	0.896		0.96(0.78-1.18)	0.692		1.21(0.87-1.70)	0.260
Dom		1.10(0.86-1.41)	0.460		1.00(0.76-1.32)	0.988		1.00(0.81-1.24)	0.964		1.20(0.84-1.72)	0.313
Rec		0.21(0.03-1.76)	0.151		0.67(0.21-2.16)	0.507		0.22(0.06-0.82)	0.024		2.05(0.37-11.29)	0.409

^a^adjusted for age, sex smoking and alcohol drinking where appropriate, Add additive model, Dom dominant model, Rec recessive model, OR odds ratio, CI confidence interval.

Linkage disequilibrium (LD) was found between these SNPs of *FTO* (D' = 0.98, r^2^ = 0.94). A haplotype analysis was carried out, and the rs9939609A-rs8050136C haplotype significantly increased the risk of TB (OR = 6.09, 95% CI: 3.27-12.34) (Table 6).

**Table 6:** **Haplotype analysis of rs9939609 and rs8050136**

Haplotype^*^	Case	Control	OR (95% CI)	***P***
	n (%)	n (%)		
TC	2768 (87.43)	2770 (89.70)	1	
AA	324 (10.23)	301 (9.75)	1.08 (0.91-1.28)	0.379
AC	73 (2.31)	12 (0.39)	6.09 (3.27-12.34)	<0.001
TA	1 (0.03)	5 (0.16)	0.20 (0.004-1.79)	0.219

^*^rs9939609-rs8050136.

To determine the potential functional effect of the SNP, we used the prediction tools SNPinfo (http://snpinfo.niehs.nih.gov/snpinfo/snpfunc.htm) and Regulome DB (http://www.regulomedb.org/). The RegulomeDB showed that rs8050136 was the binding site for the transcription factor (TF) EP300. The RegulomeDB score was 4 (supporting data: TF binding + DNase peak). No data were provided for the functional prediction of rs9939609.

## Discussion

This case-control study investigated the association between genetic polymorphisms of the *FTO* gene and tuberculosis in a Chinese Han population. This is the first study, to our knowledge, revealing the effect of genetic variations of intron 1 SNP rs9939609 of *FTO* on the risk of tuberculosis.

Immunocompetence is dependent on nutritional status and can be negatively affected by malnutrition and related disorders, including obesity. Epidemiological data support the hypothesis that obesity can affect immune function in humans [9]. Obesity has been shown to be influenced by genetic determinants. In 2007, the first common genetic variant in the *FTO* gene was identified. *FTO* is a large gene with nine exons spanning more than 400 kb on chromosome 16, and all of the SNPs identified so far are located in the first and largest intron of the gene [36]. *FTO* is predominantly expressed in the brain, particularly in the hypothalamus, and plays a key role in the control of energy homeostasis [37]. In studies with mice, the expression of *FTO* in the arcuate nucleus of hypothalamus decreased following a 48-hour fast and increased after 10 weeks of exposure to a high-fat diet [38],[39]. In humans, *FTO* appears to be essential for the normal development of major organ systems, such as the central nervous and cardiovascular system [36]. The first SNP strongly associated with BMI and obesity was located in the *FTO* gene, and this has been subsequently replicated in multiple populations [25]. Since Fralying et al. found that the variant of rs9939609 could increase the risk of developing obesity by 31%, numerous studies covering multiple populations subsequently focused on this SNP [40]-[42]. Li et al. genotyped rs9939609 in a Chinese population and found that the A allele was strongly associated with obesity and overweight [43]. The odds ratios for the allele A vs. T were 1.447 for obesity vs. normal weight (95% CI: 1.104-1.896) and 1.363 for overweight vs. normal weight (95% CI: 1.149-1.617) [43]. Xi et al. genotyped 11 SNPs of *FTO* gene in the Chinese children (6-18 years) and replicated the association of *FTO* rs9939609 with the risk of central obesity (OR:1.29, 95% CI:1.10-1.50) [40]. The association between rs9939609 polymorphism and obesity was also supported by other studies in China [29],[41],[42]. Though results of our present study do not directly support the role of *FTO* in obesity risk, it is plausible that its effect on tuberculosis occurs through obesity-related immunocompetence.

In addition to obesity, the *FTO* rs9939609 polymorphism was found to be associated with type 2 diabetes in Vietnamese (OR per A allele = 1.61, 95% CI: 1.06-2.44) [44], South Asians (OR per A allele = 1.18, 95% CI: 1.07-1.30) [45], and Chinese (OR for allele A = 1.31, 95% CI: 1.10-1.55) [43]. Interestingly, there is growing evidence regarding diabetes as a risk factor for tuberculosis, and it might affect a patient's disease presentation and treatment response [4],[46],[47]. Uncontrolled diabetics seem to have more cavities and higher positive smear rates and to show a lack of culture conversion after two months of antituberculosis therapy [48]. At the public health level, the diabetes epidemic could be one reason why efforts to reduce the global incidence of tuberculosis are having little effect, despite the high case detection rates and cure rates [47].

In this study, a LD was found between rs9939609 and rs8050136. The haplotype rs9939609A-rs8050136C was related with an increased risk of tuberculosis. Previous studies have revealed that individuals who carry a particular SNP allele at one site often predictably carry specific alleles at other nearby sites. This correlation is known as LD and a particular combination of alleles along a chromosome is termed as the haplotype [49]. The relationship between causal mutations and the haplotypes have been regarded as a tool for genetic researches----first finding association to a haplotype, and then subsequently identifying the causal mutations that it carries [49].

In the present study, we observed the role of common *FTO* rs9939609 SNP in the risk of tuberculosis, which supports the hypothesis that genetic polymorphisms of the *FTO* gene affect the host's susceptibility to infectious diseases. We believe that the effect of *FTO*-rs9939609 on the risk of tuberculosis may not solely be attributed to its role in the risk of obesity. The altered human immune function related to these genetic variants can explain, at least in part, why some people resist tuberculosis infection more successfully than others [50],[51]. This finding may help distinguish individuals who are at higher risks for tuberculosis and implement effective interventions to prevent and control the disease.

HWE test is commonly used as an initial quality check procedure in genetic association studies. Departure from HWE (DHWE) may suggest a higher risk of genotyping errors [52]. In the current study, HWE was observed among controls for either the SNP rs9939609 or the SNP rs8050136, but the P value from classical goodness-of-fit test was less than 0.05 in cases. In general, the HWE test assumes that the genotypes are sampled from the general population, and therefore the HWE tests are performed based on the controls. If the entire population is in perfect HWE, the presence of a genetic association implies that neither cases nor controls can be in HWE. Because the proportion of affected subjects in a population is small, the degree of DHWE is expected to be stronger in cases than in controls. Therefore, as an indicator of genotyping quality, compatibility with HWE should be investigated in control groups only [53]. In cohort studies, no DHWE is expected, and therefore the entire sample should be genotyped for testing HWE [53]. Recently, some authors have shown that when the disease is common in the general population, the controls do not accurately represent the general population. Therefore, using only controls for HWE testing can result in highly inflated type I error and might lead investigators to discard potential causal SNPs [52]. Wittke-Thompson et al. suggest that if a DHWE in cases or in both cases and controls is detected, it does not necessarily imply genotyping errors [54]. Rather than discarding the data, the underlying disease-genotype association should be investigated. The association may explain the observed DHWE. If not, other possible explanations such as "genotyping error, chance, and failure of assumptions underlying HWE expectations" should be explored [55]. To detect genotyping errors, repeated genotyping of the same samples is preferable over HWE testing. In the current study, we allocated DNA samples of cases and controls in each plate with no discrepancies between amplification reaction conditions. We randomly selected 5% samples to re-genotype and the consistent rate was more than 99%. We also randomly selected samples with different genotypes for gene sequencing and the consistent rate was 100%. Thus we believe that the DHWE in cases can't be attributed to the genotyping errors.

There are several limitations in this study. First, due to a case-control study design, BMI prior to the diagnosis of tuberculosis was absent, which restricted an in-depth analysis of the ancillary effect of *FTO* variants on obesity. Second, we only selected two SNPs in the *FTO* gene. Other polymorphisms and their interactions with environmental factors should also be considered [56]. Third, rs9939609 is located in the intron region of the *FTO* gene and may not be the true causal variant. Based on the RegulomeDB database, we observed a minimal binding evidence of rs8050136, which has a strong LD with rs9939609. We hypothesize that the SNP rs9939609 may be in linkage with the true functional SNP(s). Further work with both knockout and overexpression models of *FTO* and neighboring genes is likely to provide the most fruitful approach to understand the mechanisms and pathways through which these variants influence the risk of tuberculosis.

## Conclusions

Taken together, our results suggest that the *FTO* rs9939609 genetic polymorphism and the haplotype (rs9939609A-rs8050136C) are involved in the risk of tuberculosis in the Chinese population. Future studies should include a comprehensive sequencing analysis to identify the specific causative sequence variants underlying the observed associations. In future studies, nutritional status and environmental factors will be essential for elucidating gene-environment interactions.

## Authors' contributions

YF and JW conceived the idea. YF, FW, HP, SQ, JL, LW, CL and JW were involved in data collection and genotyping. YF and JW participated in the data analysis and drafted the manuscript. All authors read and approved the final manuscript.
